# A novel organic semiconductor 4-phenylthiazol-2-yl-(phenylhydrazono) acetonitrile (PTPA) thin films: synthesis, optical and electrical properties

**DOI:** 10.1038/s41598-023-39027-3

**Published:** 2023-08-10

**Authors:** Amr Attia Abuelwafa, Sahar Elnobi, M. Amélia Santos, Hesham M. Alsoghier

**Affiliations:** 1 Nano & Thin Film Lab, Physics Department, Faculty of Science, South Valley University, Qena, 83523 Egypt; 2 Physics Department, Faculty of Science, South Valley University, Qena, 83523 Egypt; 3 Centro de Química Estrutural, Instituto Superior Técnico, Universidade de Lisboa, Av. Rovisco Pais 1, 1049-001 Lisboa, Portugal; 4 Chemistry Department, Faculty of Science, South Valley University, Qena, 83523 Egypt

**Keywords:** Optical physics, Semiconductors

## Abstract

In this study, 4-phenylthiazol-2-yl-(phenylhydrazono) acetonitrile (PTPA) azo dye was synthesized and studied from optical and electrical point of view. The tautomerization phenomenon of the PTPA dye was clarified using one-dimensional (1D) and two-dimensional (2D) nuclear magnetic resonance (^1^HNMR and ^13^C NMR), absorbance (UV-Vis), emission, and Fourier transform infrared spectroscopy (FT-IR). X-ray diffraction (XRD) evaluations were indicated that PTPA in powder and thin films crystallizes in a monoclinic system structure with nonstructural characteristics. Spectrophotometric measurements of absorbance A (λ), transmittance T (λ) and reflectance R (λ) at normal incidence light in the wavelength range 200–2500 nm were used to determine the optical band gap, extinction coefficient, ***k*** and refractive index, ***n***. Also, non-linear optical parameters such as the third order non-linear susceptibility, **χ**^**(3)**^ and nonlinear refractive index, **n**^**(2)**^ of PTPA have revealed an awe-inspiring switching behavior, implying the possibility of using PTPA in optical switching systems. Finally, the electrical conductivity of the PTPA was shown to increase with rising temperature, indicating that it is a typical organic semiconductor. Mott’s parameters were determined and discussed at low temperatures. Thus, PTPA is a promising organic semiconductor with broad utility potential in organic electronics such as organic light-emitting diodes (OLEDs).

## Introduction

Organic semiconductors (OSCs) are becoming more widespread due to their enormous advantages over their inorganic counterparts. For example, they are light, easy to make, processed at low temperatures, mechanically flexible, low cost, and abundantly available^1–5^. Additionally, due to their distinguishable qualities, OSCs have lately been used to manufacture numerous high-performance electronic technology products, including solar devices, organic sensors, nonlinear electrical conductors, digital technology, memory devices, and lasers^3–7^. On the other hand, organic semiconductors have some disadvantages, such as limited electrical mobility and the instability of organic thin films during fabrication, storage, and operation due to their reactions with water and oxygen^8^. Therefore, novel OSCs with high chemical and thermal stability are required for future scale-up of the manufacturing process.

Commonly, azo dyes are the chief and most diversified category of synthetic organic chemicals used as colorants for inks, paints, toners, and laser and copier photoreceptors^9–13^. Azo dyes stand out for their simplicity in synthesis, widespread availability, low cost, chemical stability, and wide range of luminescence efficiencies. Moreover, azo dyes have solution processability, a diverse spectrum of absorption bands, fascinating electro-optical characteristics, and the ability to change their spectroscopic and electrical properties^9–16^. These features make azo dyes an encouraging candidate and a promising material with a broad spectrum of essential applications for optoelectronic applications such as solar cells^17^.

Many efforts have been made to synthesize OSCs with high nonlinear optical (NLO) properties for photonics and optoelectronics applications, such as optical limiting and switching^9,10^. Azo materials have a wide range of nonlinear susceptibility because they are usually conjugated systems with dipolar donor (D)-acceptor (A) structures^9,10,12^. This makes them useful for NLO devices.

Thiazole azo dyes have lately been employed as building blocks in a wide range of applications, including LCDs^11,13^ NLO devices^9,14,15^, and DSSCs^9,11–17^. These electrical applications are caused by the tautomerism and structural changes of dyes^18–20^.

Herein, we discovered the structure opacity by practical investigation of the tautomerism of 4-phenylthiazol-2-yl-(phenylhydrazono) acetonitrile (PTPA) azo dye in solution. In particular 1D and 2D ^1^H and ^13^C NMR spectroscopy allowed the determination of predominant PTPA conformational structure in a nonpolar solvent.

The proper application of PTPA as a thin film depends on our comprehensive knowledge of its physical characteristics. As a result, in this study, high-quality PTPA thin films were created utilizing the spin coating approach in order to investigate the detailed structural, optical, and electrical characteristics of PTPA thin films. The structural characteristics of the PTPA in powder and thin films form were examined using XRD. The Wemple and DiDomenico (single oscillator) model was also used to examine and discuss the dispersion of the refractive index. While linear and NLO properties were studied in the 200–2500 nm spectral range. Furthermore, we evaluated dc electrical conduction for PTPA in order to verify the semiconducting capabilities of PTPA thin films. So, this study will provide validation for the PTPA dye in the domain of optoelectronic devices due to its unique properties.

## Materials and methods

### Preparation

#### Synthesis of 4-phenylthiazol-2-yl-(phenylhydrazono) acetonitrile (PTPA) azo dye

**Scheme 1:**
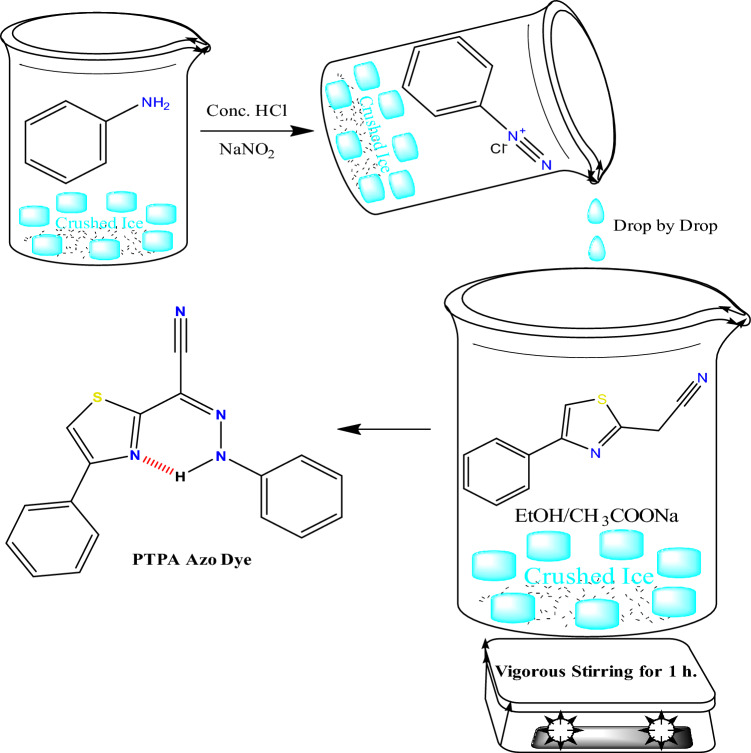
Synthesis of 4-phenylthiazol-2-yl-(phenylhydrazono) acetonitrile (PTPA) azo dye.

The PTPA dye (Scheme 1) was prepared using the method reported in the literature^9,21^. Summarizing, the diazotized aniline solution was dropwise added to an ethanolic solution of (4-phenylthiazol-2-yl) acetonitrile (0.01 M) in the presence of CH_3_COONa (0.01 M) in an ice bath for one hour with vigorous stirring.

#### Preparation of PTPA thin films

The prepared PTPA azo dye thin films were deposited onto a clean quartz substrate by spin coating technique using a chloroformed solution (20 mg/ml). The thickness of the thin films (100 and 190 nm) was estimated using the Alpha-Step IQ profilometer.

### Methods

#### NMR spectroscopy measurements

The Bruker AvanceII+ 400 MHz, 9.4 T, Ultrashielded ^1^H frequency: 400 MHz spectrometers were used to obtain the ^1^H and ^13^C NMR spectra, which operated at 400.13 MHz for ^1^H and 100.62 MHz for ^13^C. ^1^H and ^13^C chemical shifts (δ) are given in ppm relative to the solvent (CDCl_3_) main peaks (δ= 7.26 (^1^H) and 77.16 (^13^C)). The manufacturer's software (TOPSPIN 2.1) was used for all 2D tests (COSY, HSQC, and HMBC). All measurements were taken at room temperature.

#### FT-IR, UV–visible and fluorescence spectroscopy measurements

FT-IR spectrometer (JASCO (FT/IR-4100) was monitoring the infrared spectra of PTPA dye in the KBr disk. A Shimadzu 2401PC spectrophotometer was used to censor the UV-Visible absorption spectrum in wavelength range 200 to 800 nm using thermostated (*T = 298K*) 1cm matched quartz cells. The UV-Visible spectrum of 2.56 × 10^–5^ M solution of PTPA dye in CHCl_3_ (99.0–99.4% BDH AnalaR) was measured. Emission spectra of the same solution for UV-Visible absorption spectra were measured using a JASCO spectrofluorometer FP-8500 at 400 nm excitation wavelength.

#### Computational calculations

The semiempirical modeling of the PTPA structure (Scheme 1) was attained using PM6 and PM6/CI (configuration interaction) with optimization in the ground (S_0_) and first excited (S_1_) states, respectively. The PM6 (COSMO) model in chloroform was used to investigate this dye's tautomerism. The geometric structure of this dye’s *Z*-hydrazone form was created by eliminating the methyl group from a single crystallographic structure with the deposition number (617431). All geometry calculations were performed on a personal computer using a MOPAC 2009-free license obtained from the internet. A further DFT geometric calculation was completed using Gaussian 03 program via B3LYP method. Input files of PTPA molecule were fixed with Gauss View 5.0.9 in gases state. A full optimization was performed up to a higher basis set 6-31G++ (d, p).

#### Cyclic voltammetry measurements

Cyclic voltammetry (CV) measurements were achieved using a stock solution of 0.0033 g of PTPA in 5 mL of MeOH with a drop of DMF. 400 μL of the stock was added to a 20-mL Britton-Robinson buffer (BRB) solution at a pH of 2.5. Versa STAT4 Versa STAT 4 was also utilized to carry out the electrochemical tests, which were linked to a three-electrode system cell with a carbon paste electrode (CPE) of 0.3 mm diameter as the working electrode, Ag/AgCl as the reference electrode, and Pt wire as the counter electrode.

#### XRD and optical measurements of thin films

Rigaku RINT 2100 diffractometer was used to record XRD patterns of PTPA azo dye in powder and thin films form. The optical measurement films were recorded at normal incidence using spectrophotometer in the wavelength range of 200–2500 nm (JASCO model V-570 UV-Vis-NIR).

#### Dc electrical measurements of thin films

Dc electrical measurements were carried out in a flat configuration with Au electrodes spaced 5 mm apart (see Scheme S1). The DC electrical conductivity of PTPA films was evaluated by measuring resistance as a function of temperature using a Keithley 2420 instrument throughout a temperature range (300–450 K). The resistivity was calculated as follows^2^:

1 $$\uprho ={\mathrm{R}}_{e} \frac{(wd)}{L},$$

where, ${{\varvec{R}}}_{{\varvec{e}}}$ represents the electrical resistance**, *w*** represents the width of the film, ***d*** represents the thickness of the film, and ***L*** is the length of the film.

## Results and discussion

A common phenomenon in azo dyes under investigation is azo-hydrazone tautomerism^22–24^. Analyzing azo-hydrazone tautomerism in azo-dye solutions is made possible by NMR spectroscopy in 1D and 2D spectroscopy^24–28^.

### 1D and 2D ^1^H and ^13^C NMR of PTPA

**Figure 1:**
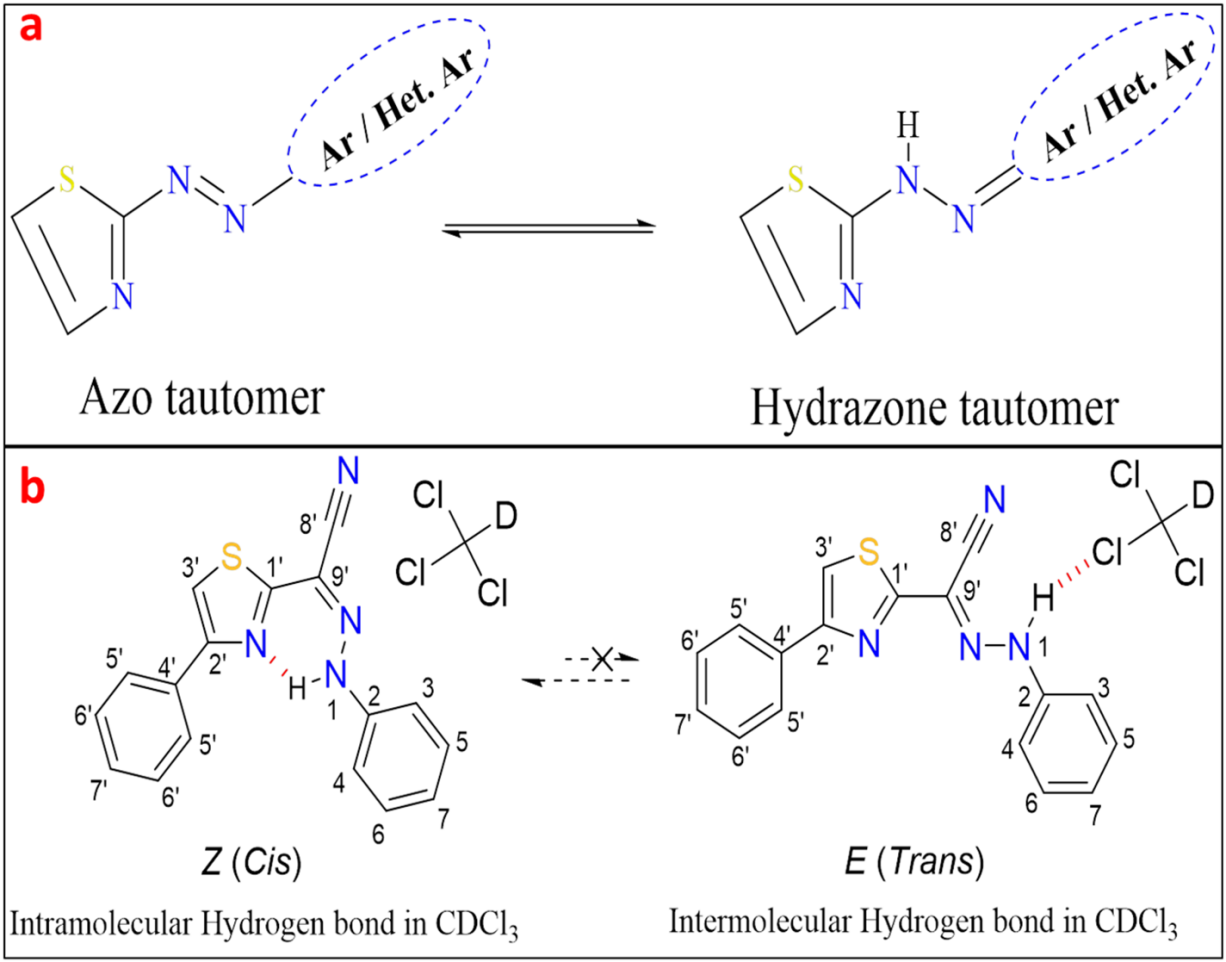
(**a**) Azo-Hydrazone tautomerism of thiazole azo dye. (**b**) *The intra and intermolecular hydrogen bonds of the predominant *Z*- and *E-* hydrazone forms of PTPA dye. (*Numbering of carbons and protons will not be continuous and not follow numbering rules, where the thiazolylacetonitrile moiety has numbers from 1' to 9’, on the other hand the substituted phenyl ring has numbers from 1 to 7).

**Figure 2:**
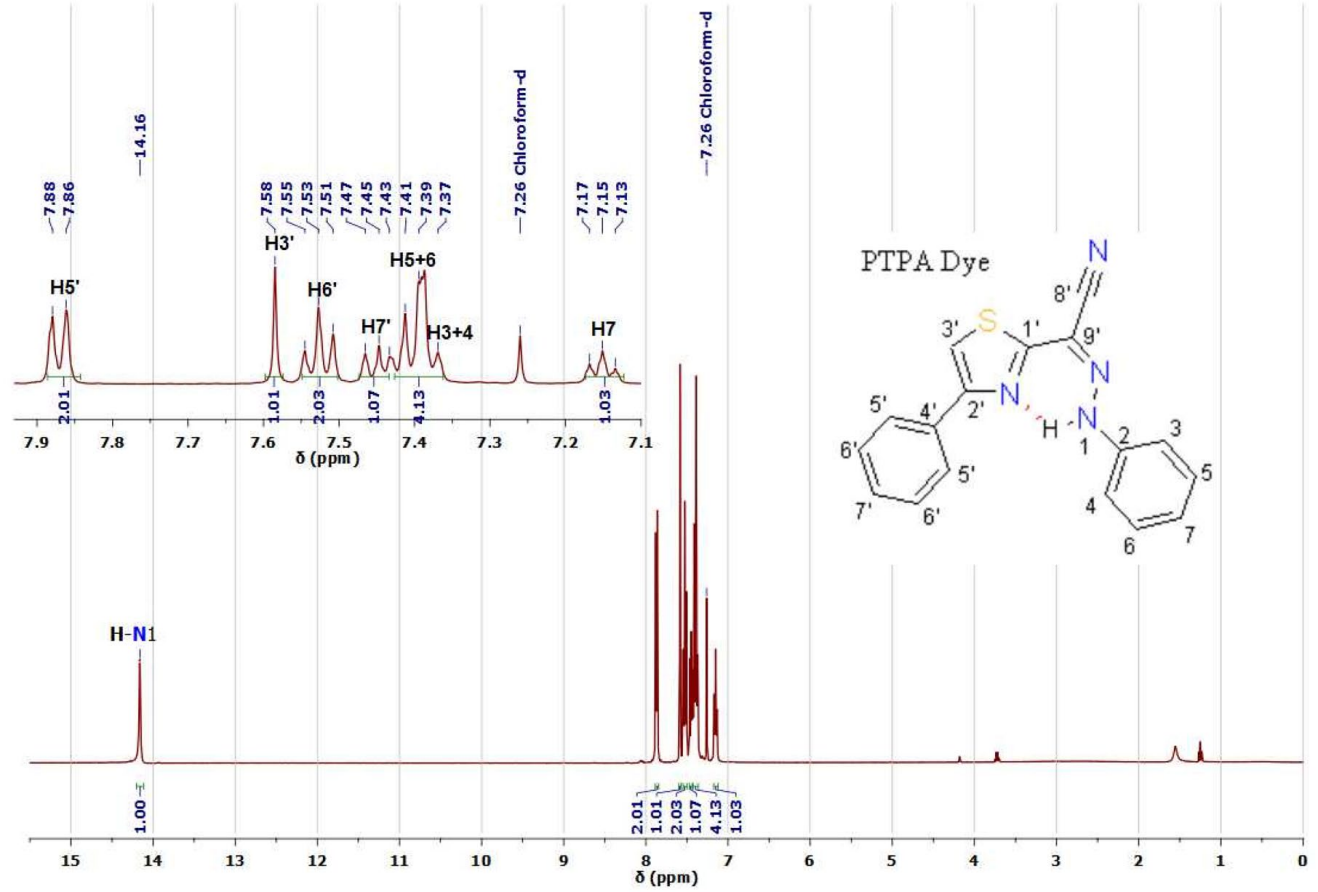
^1^H NMR spectrum of 4-phenylthiazol-2-yl-(phenylhydrazono) acetonitrile (PTPA) azo dye in CDCl_3_.

According to computational calculations and UV-Visible spectra in various solvents^22,23^, thiazole azo dyes are formed in tautomeric equilibrium between azo and hydrazone forms, as illustrated in Fig. 1a^22–26^. Though, 1D and 2D ^1^H&^13^C NMR investigations of 4-phenylthiazol-2-yl-(phenylhydrazono) acetonitrile (PTPA) dye in CDCl_3_ solution reveal that this dye is often present in the Z geometric hydrazone configuration (see Fig. 1b). This point is the result of an intramolecular hydrogen connection between the hydrazone (H-N1) proton and the nitrogen atom (N) in thiazole^20^. Further, the intramolecular hydrogen bond is the primary source of the hydrazone (H-N1) proton's strong downfield chemical shift (δ= 14.16 ppm) (see Fig. 2 and Table S1)^24,25,29,30^. 1D C^13^ (Figs. S1, S2, S3), 2D COSY (Fig. S4), HSQC (Fig. S5), and HMBC experiments^25,31^ are used to determine ^1^H chemical modifications, protonated carbons, and quaternary carbons, as well as the predominant tautomeric form in solutions (see Table S1).

**Figure 3:**
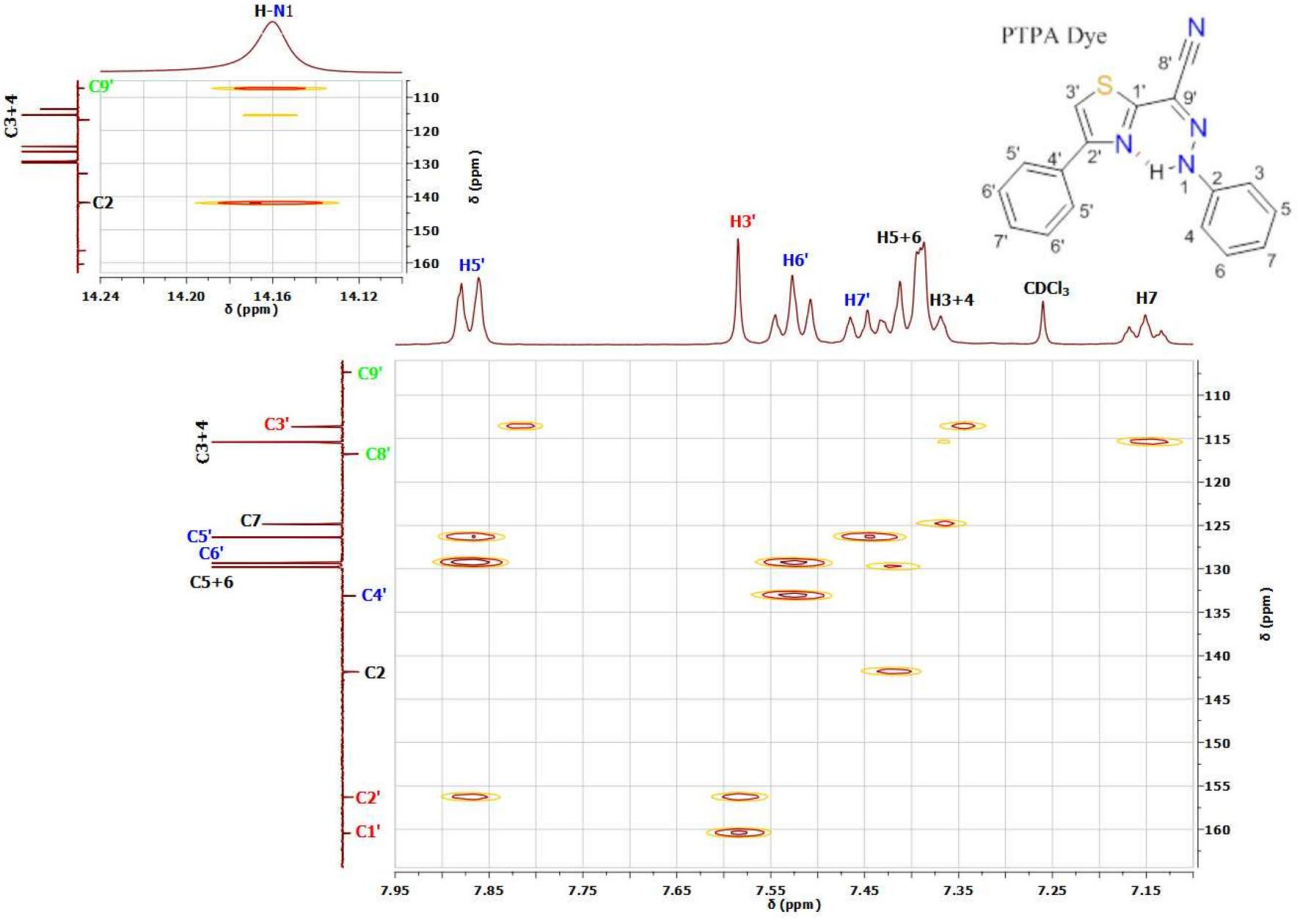
HMBC spectrum of 4-phenylthiazol-2-yl-(phenylhydrazono) acetonitrile (PTPA) azo dye in CDCl_3_; HMBC spectrum of hydrazone (H-N1) proton of this dye in CDCl_3_ (inserted).

The HMBC spectra of PTPA dye in CDCl_3_ are revealed a link between the hydrazone (H-N1) proton (δ= 14.16 ppm) and the ortho-C3, 4 atoms (δ= 115.42 ppm), the C2 atom (δ= 141.87 ppm) in the phenyl moiety (δ= 107.34 ppm) (Fig. 3, Table S1). HMBC results demonstrated unequivocally that the primary geometrical structure of PTPA dye is Z-hydrazone form augmented by an intramolecular hydrogen link between the hydrazone (H-N1) proton and nitrogen atom (N) in the phenylthiazole moiety^20^.

### Analysis of absorbance, emission, and FT-IR Spectra

**Figure 4:**
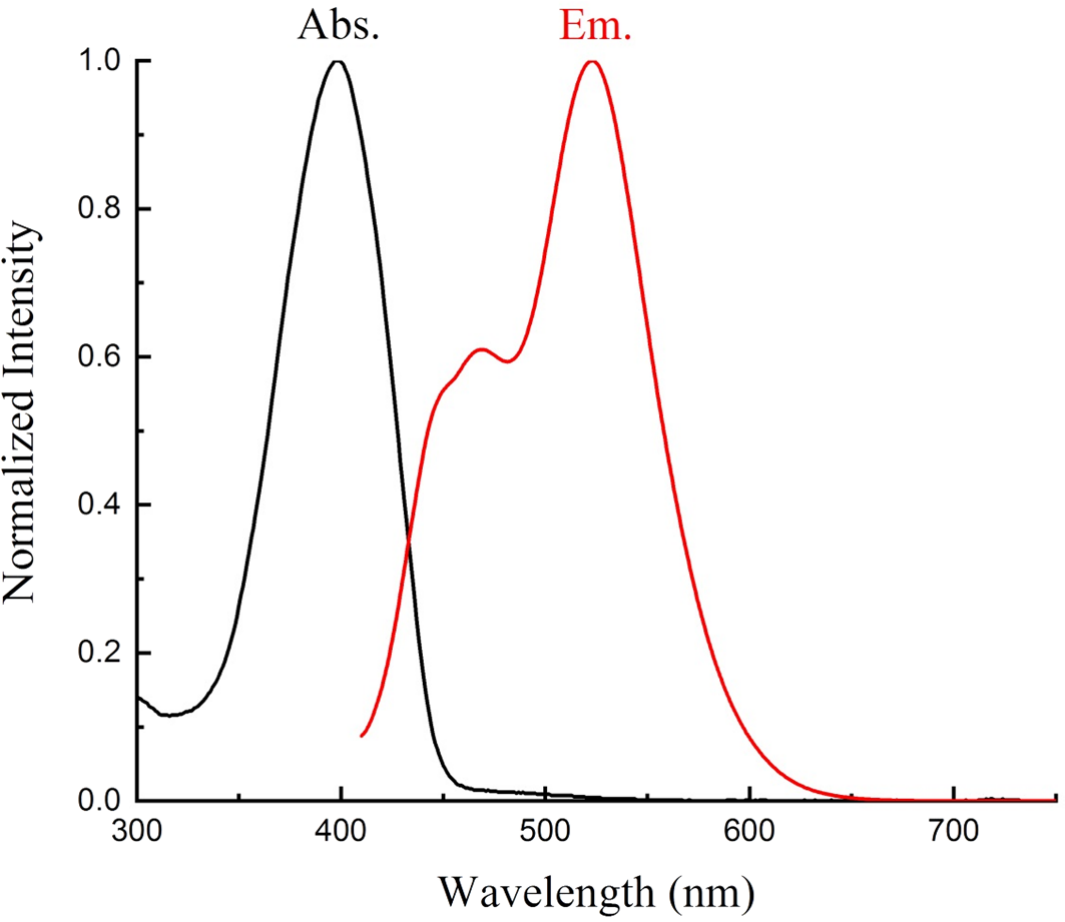
Normalized absorption (Abs.) and emission (Em.) spectra for the dye (**PTPA**) in CHCl_3_.

**Figure 5:**
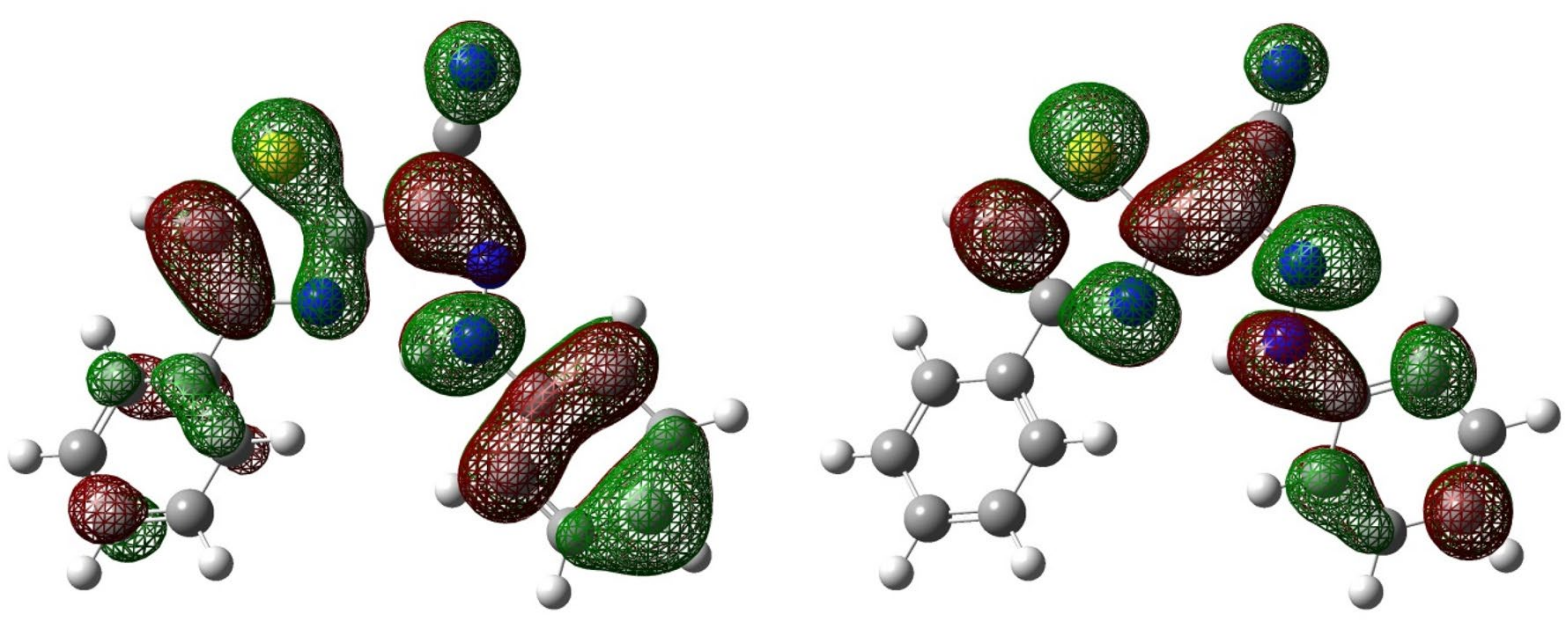
HOMO (left) and LUMO (right) of PTPA calculated at DFT/B3LYP/6-31++G (d,p).

The realistic optical band gap (${\mathrm{E}}_{\mathrm{g}}^{\mathrm{Opt}}$) of the examined PTPA dye is computed from the maximum wavelength (Fig. 4) of the absorption band (λ_max_ = 398.5 nm). However, the predicted energy difference ∆E_S1-S0_ between the excited and ground states of *Z*-hydrazone form in PM6 and PM6 COSMO simulations is 3.183 eV and 3.145 eV, respectively. But, the (∆E_LUMO-HOMO_) value of the PTPA resulting from DFT gas state optimization is 3.428 eV. The experimental ${\mathrm{E}}_{\mathrm{g}}^{\mathrm{Opt}}$ agrees with the theoretically calculated (∆E_S1-S0_) energy difference. This allows the absorption band at λ_max_= 398.5 nm to be generated by an intramolecular charge transfer (ICT) π S_0_→π* S_1_transition involving the entire-electronic system^32–34^ between the phenyl donating moiety and the thiazole acceptor ring, as shown in the HOMO and LUMO orbitals in Fig. 5. The active electronic density shift from the phenyl-donating ring to the acceptor 4-phenylthiazole is seen in this HOMO and LUMO orbital diagram.

According to NMR measurements, PTPA azo dye mostly exists in a single tautomeric form (Z-hydrazone) as seen by the strong absorption band at max= 398.5 nm ( see Fig. 4). Although this dye has a wide emission band at λ_em_ = 526 nm and a shoulder at λ_em_ = 478 nm, it have more than one structural form present in the excited state. FT-IR spectrum (see Fig. S6) of PTPA dye shows distinguishable bands at 3410 (N–H), 3100–2840 (C–H_Aromatic_), 2213 (C≡N), 1601 (N=C), 1544 (C=N–N–), 1477-1260 (C=C_Aromatic_), 1084 (C-N), and 710 cm^-1^ (C–S). These bands confirm the suggested *Z*-hydrazone chemical structure of PTPA dye.

### XRD studies for PTPA thin films

**Figure 6:**
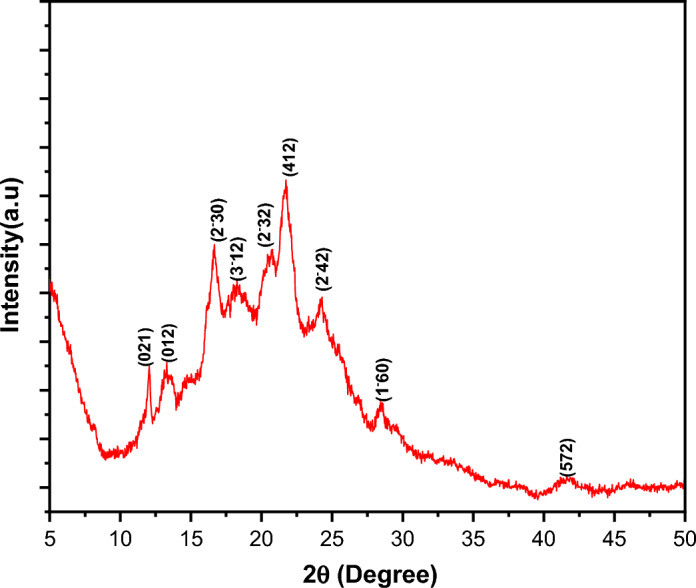
XRD pattern for PTPA powder.

**Figure 7:**
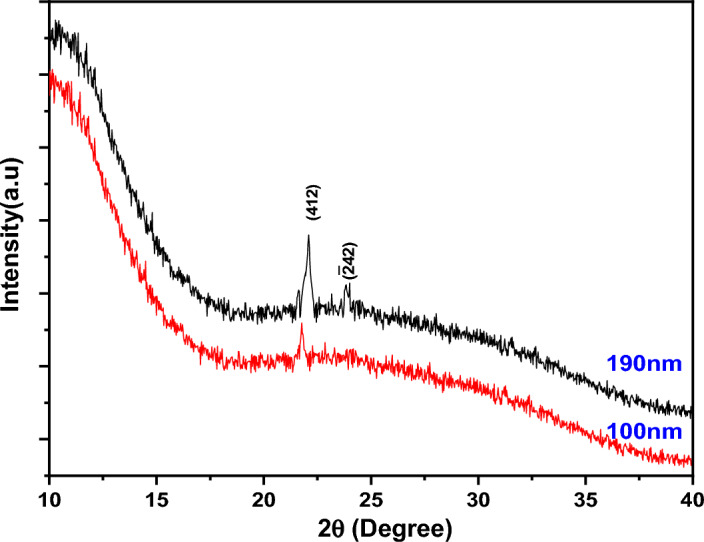
XRD patterns for PTPA thin films.

The XRD pattern of PTPA in powder form is shown in Fig. 6. The existence of numerous diffraction peaks of varying strengths in the pattern indicates that the PTPA powder is polycrystalline. The computer programs CHECKCELL and CRYSFIRE are used to determine the Miller indices unit and cell properties of PTPA^35,36^ The monoclinic phase structure of PTPA powder is P_21/c_ with a (412) preferred orientation. According to structural data, a= 21 Å, b= 22 Å, c= 14 Å, α= 90.00°, β= 91.00°, and γ= 90.00° are the lattice parameters. A similar phase structure appeared in BTPA azo dye X-ray crystallographic data^16^. Figure 7 depicts the XRD pattern of the PTPA film on the quartz substrate. Scherrer’s equation^37^ is used to estimate the average crystallite size, ***D*** as 20.36 nm for a film thickness of 100 nm and 34.56 nm for a film thickness of 190 nm. Nanometric measurements of crystallite sizes show that PTPA films have a distinguishing nanostructure.

### Optical studies of PTPA thin films

**Figure 8:**
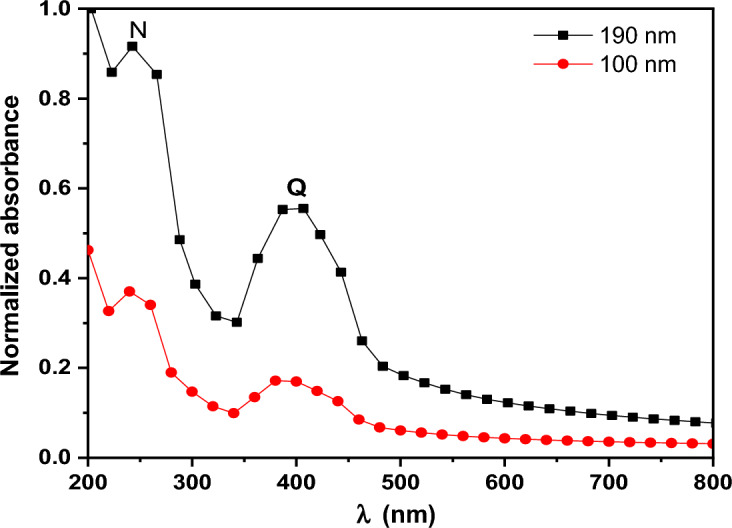
Normalized absorbance for PTPA thin films.

The absorbance spectra *A (λ)* for PTPA thin films in **UV-Vis-NIR** areas are shown in Fig. 8. The (π–π*) transition causes the main band under investigation to emerge at around 390 nm. An inherent molecular transition in PTPA (n-π*) causes the band at 239 nm. A (**λ**) can be used to calculate the absorption coefficient ($\alpha$) using the formula shown below^38,39^:

2 $$\mathrm{\alpha }=\frac{2.303\mathrm{A}(\uplambda )}{\mathrm{d}}.$$

**Figure 9:**
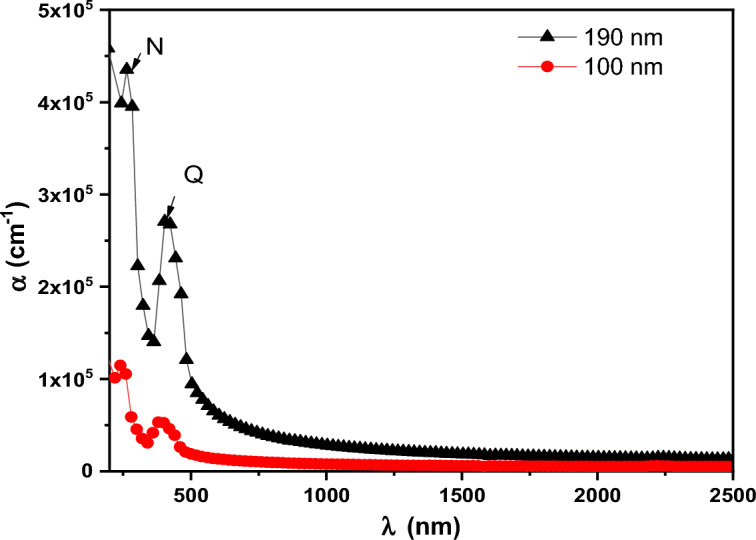
Absorption coefficient, α for PTPA thin films.

**Figure 10:**
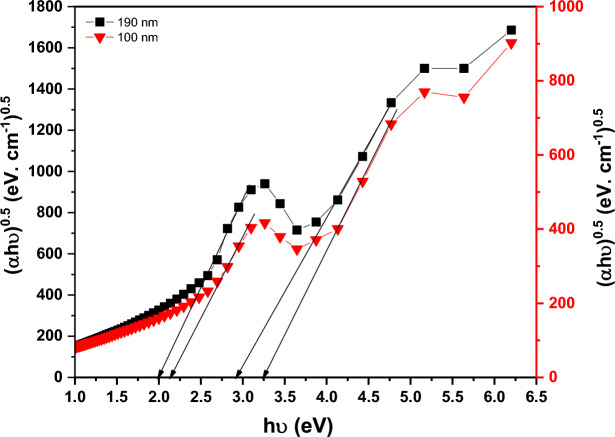
(αhν)^1/2^ vs. hν for PTPA thin films.

**Figure 11:**
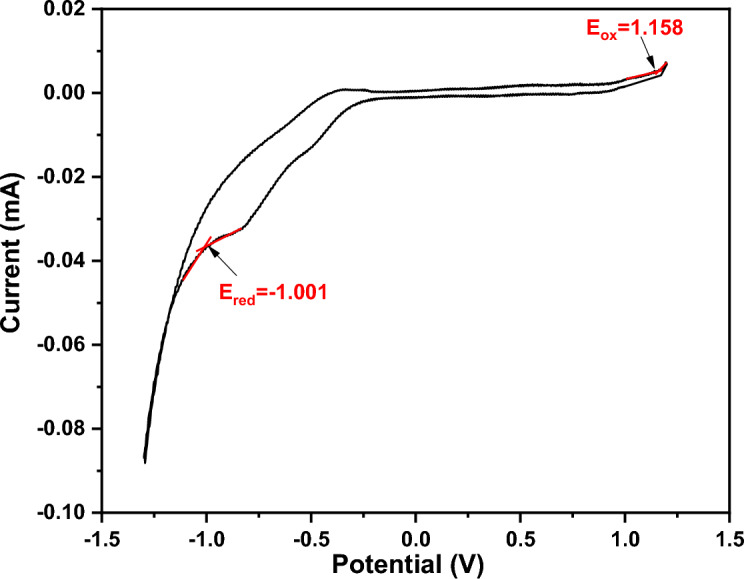
Cyclic voltammogram of PTPA.

Figure 9 depicts the ($\alpha$) of the as-deposited PTPA thin films (100 and 190 nm) with a value in the region of 10^5^ cm^-1^ in the wavelength (λ) range of 200–2500 nm. The redshifts in absorbance coefficient are caused by an increase in the crystallinity of PTPA thin films as the film thickness increases. It is common to use Tauc’s equation in many organic materials to investigate the kind of optical transitions and the magnitude of the energy gap at the basic absorption edge using the following expression^40–42^:

3 $${(\mathrm{\alpha h\nu })}^{1/S}=\mathrm{B}{\left(\mathrm{h\nu }-{\mathrm{E}}_{\mathrm{g}}^{\mathrm{Opt}}\right)}^{S},$$

where, ***S*** is a parameter that is affected by the transition probability. It is discovered that the best fit is achieved when ***S*** =2. As a result, the kind of electronic transition is an indirect permitted transition (see Fig. 10). Two band maxima in PTPA thin films indicate two indirect optical transitions between LUMOs and HUMOs states. The first energy value ${\mathrm{E}}_{\mathrm{g}1}^{\mathrm{Opt}}$ correlates to the optical absorption **Q** band, while the second energy value ${\mathrm{E}}_{\mathrm{g}2}^{\mathrm{Opt}}$ relates to the optical absorption **N**-band. ${\mathrm{E}}_{\mathrm{g}1}^{\mathrm{Opt}}$ and ${\mathrm{E}}_{\mathrm{g}2}^{\mathrm{Opt}}$ values of the PTPA thin films for two different thicknesses (100 and 190 nm) are summarized in Table 1. In accordance with Table 1, the ${\mathrm{E}}_{\mathrm{g}}^{\mathrm{Opt}}$ of PTPA thin films decrease as the film thickness increases. This decrease in ${\mathrm{E}}_{\mathrm{g}}^{\mathrm{Opt}}$ is caused by improved crystallinity and an increase in the crystallite size of the PTPA thin films (XRD findings). The HOMO/LUMO calculation was essential for modifying device architectures to achieve the proper energy levels between emitting and conveying materials, thereby averting interface emission and other device issues. Additionally, this is advantageous for enhancing device performance. HOMO and LUMO energy levels of PTPA were determined using CV (see Fig. 11). According to the equations below^43^:

4 $${\mathrm{E}}_{\mathrm{HOMO}}=-\left({\mathrm{E}}_{ox}+4.4\right)eV,$$

5 $${\mathrm{E}}_{\mathrm{LUMO}}=-\left({\mathrm{E}}_{red}+4.4\right)eV,$$

where, ${\mathbf{E}}_{{\varvec{o}}{\varvec{x}}}$ and ${\mathbf{E}}_{{\varvec{r}}{\varvec{e}}{\varvec{d}}}$ are beginnings of oxidation and reduction potentials which have values 1.158 and − 1.001 V of PTPA, respectively. HOMO-LUMO energy gap is calculated to be 2.16 eV. The energy band gap calculated from CV is extremely similar to that inferred from absorption onset, demonstrating the reliability of electrochemical examination of LUMO and HOMO energy levels.

**Table 1:** Estimated optical parameters for PTPA thin films.

Estimated parameters	Thin films thickness (nm)	
	100	190
(${\mathrm{E}}_{\mathrm{g}1}^{\mathrm{Opt}}$) eV	2.13	1.99
(${\mathrm{E}}_{\mathrm{g}2}^{\mathrm{Opt}}$) eV	3.24	2.92
(E_o_) eV	4.68	4.87
(E_d_) eV	11.52	13.86
${\varepsilon }_{\infty }^{WD}$	3.46	3.83
${n}_{o}$	1.86	1.96
${\varepsilon }_{L}$	3.66	4.05
$\frac{N}{{m}^{*}}$ ×10^55^	6.58	6.71
${\chi }^{(3)}$ × 10^–13^(hυ → 0)	2.51	4.48
${n}^{\left(2\right)}$ × 10^–12^ (hυ → 0)	5.07	8.62
${\chi }^{(3)}$ × 10^–10^(i)	4.82	5.48
${\chi }^{(3)}$ × 10^–10^ (ii)	4.21	4.85
${n}^{\left(2\right)}$ × 10^–9^ (i)	9.83	10.06
${n}^{\left(2\right)}$ × 10^–9^ (ii)	8.57	9.35
β_Cmax,_ cm/GW	32.89	42.84

**Figure 12:**
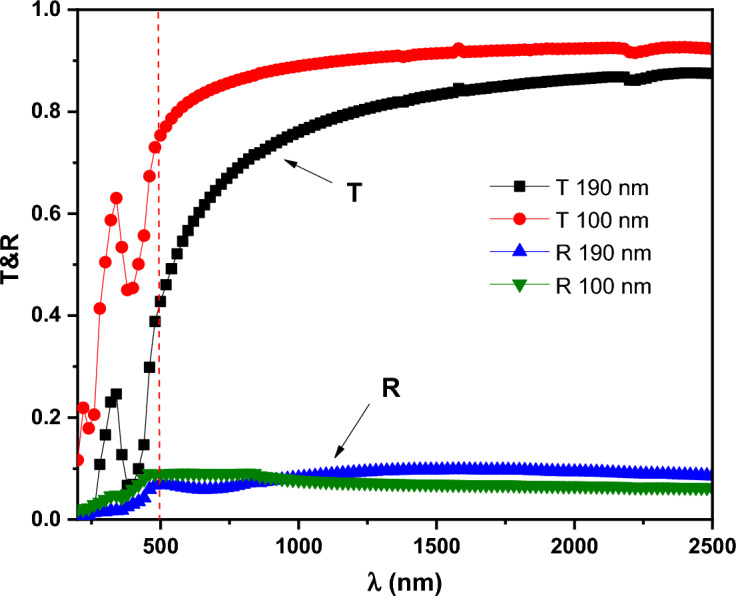
Optical transmission T (λ) and reflection R (λ) for PTPA thin films.

The distribution of T (λ) and R (λ) spectra for PTPA film in the wavelength range (200-2500 nm) for two distinct thicknesses (100 and 190 nm) are shown in Fig. 12. The absorption edge of PTPA appears in the absorption area (λ >500 nm) (2 edge “valleys” regions). However, all PTPA thin films become almost transparent in the non-absorbing region (λ > 500 nm) owing to a very little amount of lost energy due to scattering. Linear optical constants, including the refractive index, ***n*** and the extinction coefficient, ***k*** can be calculated from α (λ) and *R* (λ) as shown below^44,45^:

6 $$k=\frac{\propto \lambda }{4\pi },$$

7 $$n=\frac{1+R}{1-R}+\sqrt{\frac{4R}{{\left(1-R\right)}^{2}}-{k}^{2}.}$$

**Figure 13:**
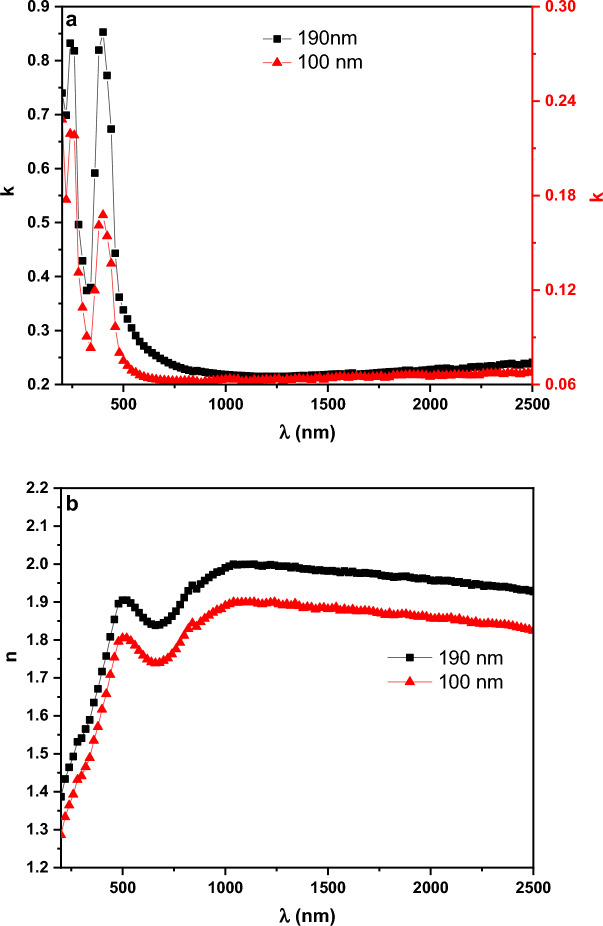
Variation of (**a**) k and (**b**) n vs. λ for PTPA thin films.

**Figure 14:**
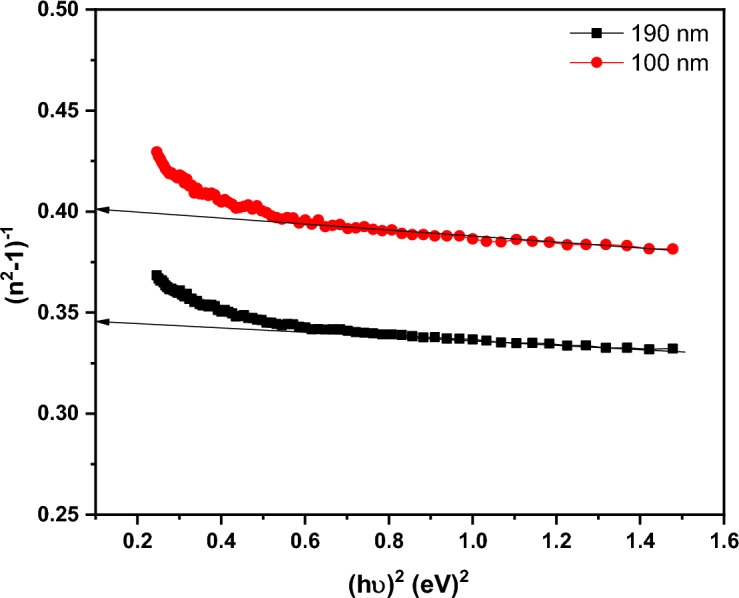
(n^2^–1)^-1^ vs. (hν)^2^ for PTPA thin films.

**Figure 15:**
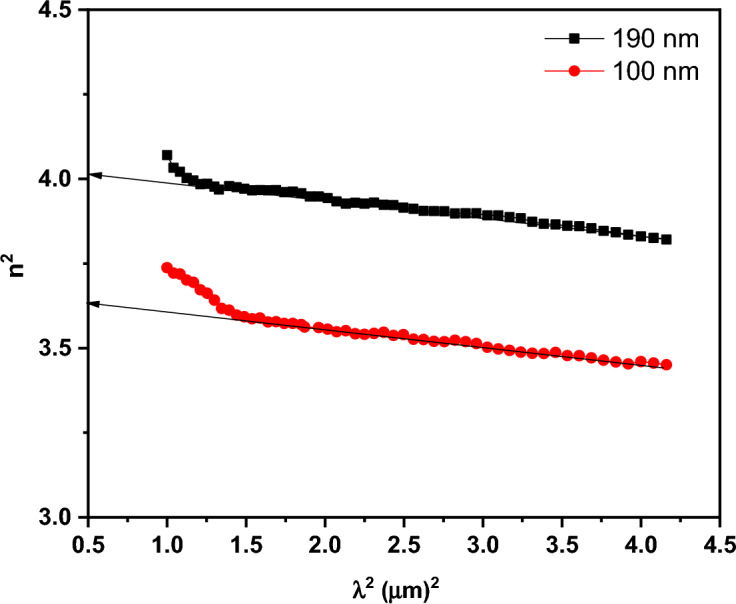
n^2^ vs. λ^2^ for PTPA thin films.

Figure 13a and b illustrates the distribution of ***k*** and ***n*** spectra for PTPA film in the wavelength range (200–2500 nm) for two different thicknesses (100 and 190 nm). The reduced light energy loss associated with thin PTPA films with low ***k*** values suggests potential use in optoelectronics (see Fig. 13a). The dispersion curve of ***n*** decreases smoothly with wavelength (normal dispersion) at wavelengths greater than 830 nm (see Fig. 13b). Using the Wemple and DiDomenico (single oscillator) method, ***n*** dispersion parameters are able to be calculated at this site with the help of the following equation^44,46^:

8 $${n}^{2}\left(\mathrm{h\nu }\right)-1=\frac{{E}_{d}{E}_{o}}{{E}_{o}^{2}-{\left(h\nu \right)}^{2}},$$

where, ${{\varvec{E}}}_{{\varvec{o}}}$ denotes oscillator energy and ${{\varvec{E}}}_{{\varvec{d}}}$ denotes dispersion energy. The values of ${{\varvec{E}}}_{{\varvec{d}}}$ and ${{\varvec{E}}}_{{\varvec{o}}}$ can be simply calculated from the slope and intercept of ${({{\varvec{n}}}^{2}-1)}^{-1}$ versus ${({\varvec{h}}{\varvec{\upsilon}})}^{2}$ (see Fig. 14). Table 1 shows the derived ${{\varvec{E}}}_{{\varvec{d}}}$ and ${{\varvec{E}}}_{{\varvec{o}}}$ values. The values of ${{\varvec{E}}}_{{\varvec{d}}}$ fall as the thickness of the films rises, whereas the values of ${E}_{o}$ grow. Extrapolating the linear component of the dispersion relationship towards h $\nu$= 0 eV yields the infinite wavelength dielectric constant ${{\varvec{\varepsilon}}}_{\boldsymbol{\infty }}$. The formula ${\mathbf{n}}_{\mathbf{o}}=\sqrt{{{\varvec{\varepsilon}}}_{\boldsymbol{\infty }}}$ can also be used to compute the static refractive index ${{\varvec{n}}}_{{\varvec{o}}}$. Table 1 summarizes the values of ${{\varvec{\varepsilon}}}_{\boldsymbol{\infty }}$ and ${{\varvec{n}}}_{{\varvec{o}}}$ for PTPA thin films. By examining the, ***n*** data in the transparent zone and using the following relation the dielectric constant at high frequency ${\varepsilon }_{L}$ and the ratio of carrier concentration to effective mass $\left(\frac{N}{{m}^{*}}\right)$ can be calculated^40,44,47^:

9 $${n}^{2}={\varepsilon }_{L}-\left(\frac{{e}^{2}}{4 {\pi }^{2} {c}^{2}{\varepsilon }_{o}}\right)\left(\frac{N}{{m}^{*}}\right){\lambda }^{2},$$

where, **e** represents the electron charge, the values of ${{\varvec{\varepsilon}}}_{{\varvec{L}}}$ and $\left(\frac{{\varvec{N}}}{{{\varvec{m}}}^{\boldsymbol{*}}}\right)$ which are computed from the intercept and the slope of ${{\varvec{n}}}^{2}$ versus ${{\varvec{\lambda}}}^{2}$ as shown in Fig. 15. Values of ${{\varvec{\varepsilon}}}_{{\varvec{L}}}$ and $\left(\frac{{\varvec{N}}}{{{\varvec{m}}}^{\boldsymbol{*}}}\right)$ are tabulated in Table 1. It is evident that the value of ***ε***_***L***_*** > ε***_***∞***_ is due to the contribution of free charge carriers to the polarisation process that occurs inside the material when light shines it^40^. Furthermore, as thickness grow, the rising values of ${{\varvec{E}}}_{{\varvec{d}}}$***,***${{\varvec{E}}}_{{\varvec{o}}}$***, ε***_***∞,***_${\boldsymbol{ }{\varvec{n}}}_{{\varvec{o}}}$***, ε***_***L***_ and $\left(\frac{{\varvec{N}}}{{{\varvec{m}}}^{\boldsymbol{*}}}\right)$ may be attributed to the decrease of lattice defects and lattice vibration.

### Nonlinear optical parameters for PTPA thin films

An empirical equation based on extended Miller's rule and linear refractive index dispersion, can be used to determine the NLO parameters, which include the third-order nonlinear susceptibility ${{\varvec{\chi}}}^{\left(3\right)}$ and. the nonlinear refractive index ${{\varvec{n}}}^{\left(2\right)}$**(**see Eqs. (10) and (11). While the nonlinear absorption coefficient (${{\varvec{\beta}}}_{{\varvec{c}}}$) can be estimated using the Sheik-Bahae model (see Eq. (12))^48–51^.

10 $${\chi }^{\left(3\right)}=1.7{\left[\frac{{E}_{o}{E}_{d}}{4\pi \left({E}_{o}^{2}-{(h\nu )}^{2}\right)}\right]}^{4}\times {10}^{-10},$$

11 $${n}^{\left(2\right)}=\frac{12\pi {\chi }^{\left(3\right)}}{{n}_{o}},$$

12 $${\beta }_{c}\left(\omega \right)=\frac{3100\sqrt{21}{\left[\left(2h\nu /{E}_{g}^{Opt}\right)-1\right]}^{3/2}}{{n}^{2} {{E}_{g}^{Opt}}^{3}{\left(2h\upsilon /{E}_{g}^{Opt}\right)}^{5}}.$$

**Figure 16:**
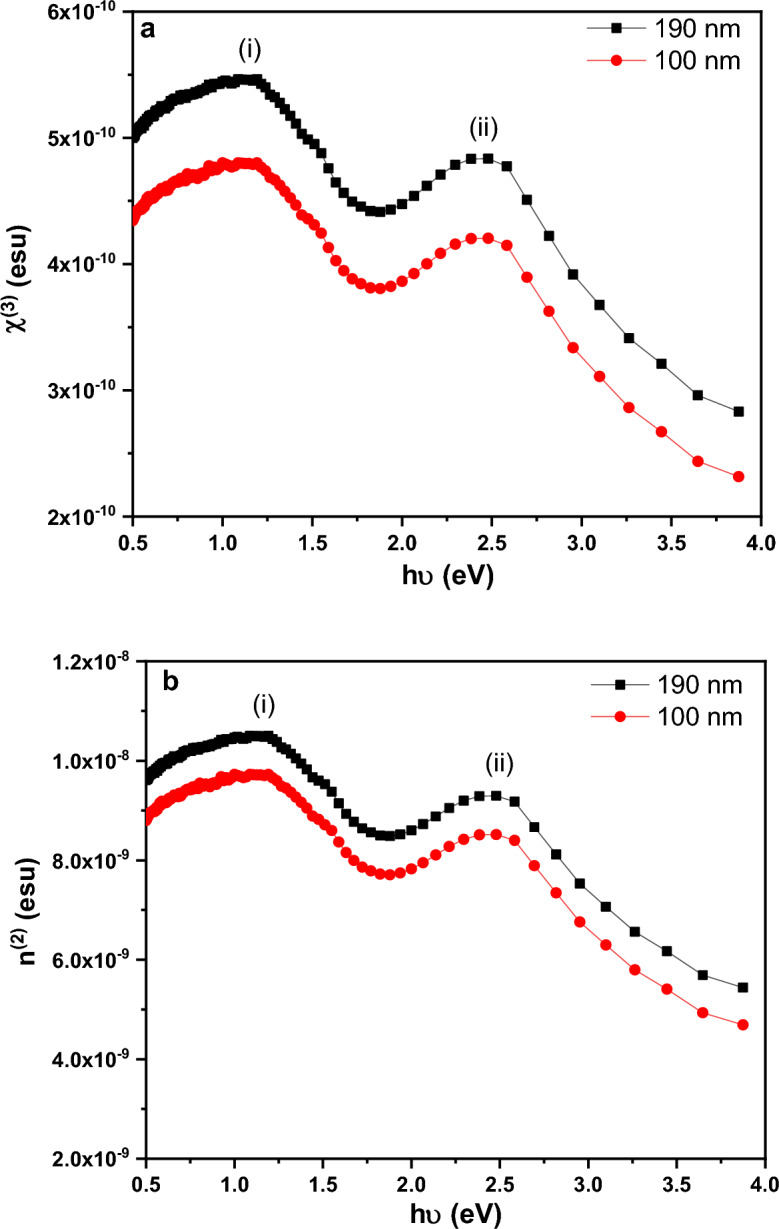
Variation of (**a**) χ^(3)^ and (**b**) n^(2)^ vs. hν for PTPA thin films.

Figure 16a and b displays the variations of ${\chi }^{(3)}$ and ${n}^{(2)}$ as a function of photon energy for as-deposited PTPA at two distinct thicknesses (100 and 190 nm). Table 1 provides the projected values of ${n}^{(2)}$ and ${\chi }^{(3)}$ (esu) when h $\nu$= 0 eV for the analyses films. The potential of optical switching using PTPA thin films is shown in Fig. 16a and b, where the films’ significant peaks are located at (i) 1.16 and (ii) 2.47 eV. Moreover, Table 1 shows values of ${\chi }^{(3)}$ and ${n}^{(2)}$ at photon energies of 1.16 and 2.47 eV for two distinct thicknesses (100 and 190 nm) of PTPA thin films. The increases in values of noticeable peaks in Figs. 16a and b are due to a drop in E_g_^opt^ as the film thickness grew. PTPA’s nonlinear characteristics are revealed an awe-inspiring switching behavior, implying the possibility of using PTPA in optical switching systems.

**Figure 17:**
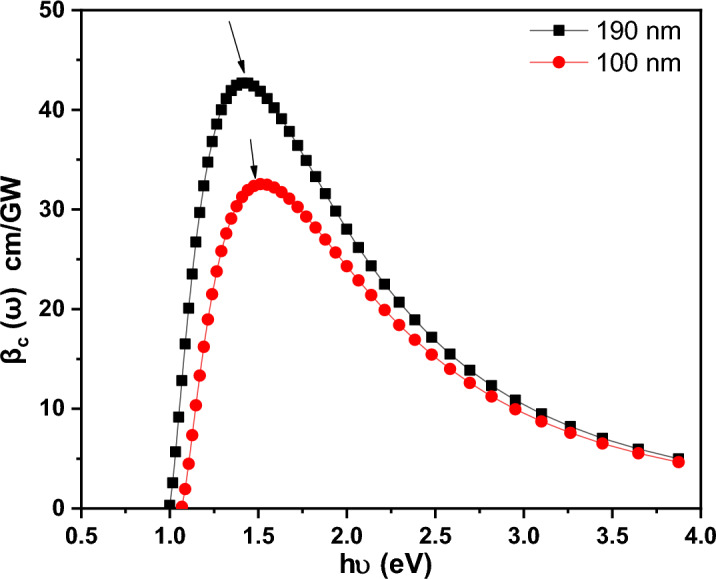
Variation of βc, vs. hν for PTPA thin films.

Changes in ***βc*** as a function of ***hv*** for PTPA thin films are shown in Fig. 17. When ***hv*** is raised, ***βc u***psurges gradually until it reaches a maximum value (see Table 1) after which it drops back down. A number of organic thin films showed comparable performance of ***βc*** with ***hv*** including NiTPP/FTO^38^, (4TFEO) 4-ZnPc/quartz^40^, CN-PPV/FTO^41^, MnTPPCl/quartz^52^, and Toluidine Blue/quartz^53^. This finding also illustrates how thickness affects the enhancement of ***βc.*** This behavior could be explained by a decrease in ${\mathrm{E}}_{\mathrm{g}}^{\mathrm{Opt}}$ in the instance of the 190 nm thin films.

### Electrical conductivity studies of PTPA thin films

**Figure 18:**
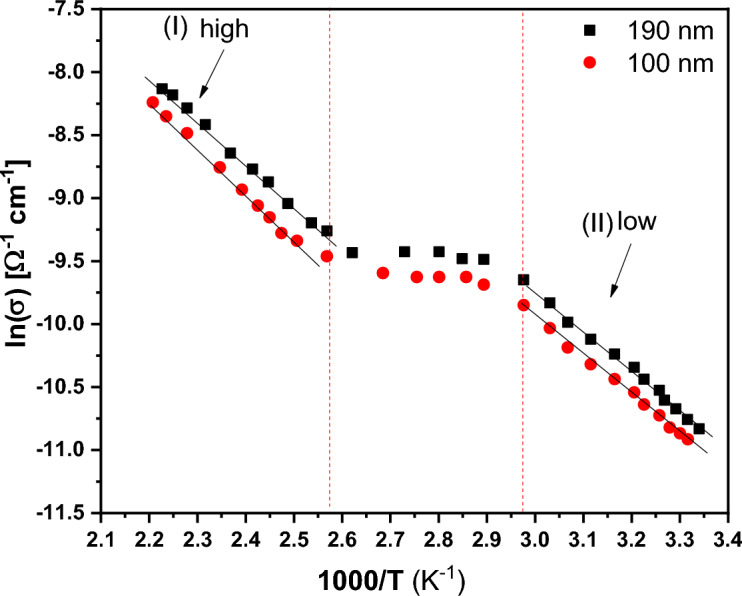
DC conductivity of PTPA thin films as a function of reciprocal temperature.

The electrical conductivity of the synthesized PTPA nanoparticles is investigated through their temperature-dependent conductivity within the range of temperatures (300-450 K). The produced PTPA thin films' dc electrical conductivity is shown in Fig. 18. It is evident from Fig. 18 that the direct current DC conductivity increases as temperature rises. As evidenced by its DC conductivity, the PTPA is a typical organic semiconductor^2^. Plots in Fig. 18 illustrate Arrhenius’s behavior in the two temperature ranges [high I and low II]^2,54–56^.

13 $${\sigma }_{dc}={\sigma }_{oI}\mathrm{exp}\left(-\frac{\Delta {\mathrm{E}}_{\sigma I}}{{k}_{B}T}\right)+{\sigma }_{oII}\mathrm{exp}\left(-\frac{\Delta {\mathrm{E}}_{\sigma II}}{{k}_{B}T}\right),$$

where, ${{\varvec{\sigma}}}_{{\varvec{d}}{\varvec{c}}}$ represents the dc electrical conductivity, ${{\varvec{\sigma}}}_{{\varvec{o}}}$ represents the zero kelvin electrical conductivity, ${{\varvec{k}}}_{{\varvec{B}}}$ represents the Boltzmann constant, ${\varvec{\Delta}}{\mathbf{E}}_{{\varvec{\sigma}}}$ represents the activation energy, and **T** represents the absolute temperature. Slope and intersection of the straight lines in the [high I and low II] parts of the plot can be used to determine the values of (**ΔE**_**σI**_**, ΔE**_**σII**_, **σ**_**oI**_**,** and **σ**_**oII**_) for all of the thin films under evaluation, as noted in Table 2. The shift in slope, and thus the variation in ${\varvec{\Delta}}{\mathbf{E}}_{{\varvec{\sigma}}}$ indicate a transition from extrinsic to intrinsic conduction. The activation energy in intrinsic semiconductors is less than half the value of the optical energy gap, which is already suitable with the determined **ΔE**_**σI**_ values for PTPA thin films. Lower temperature conduction is clarified in terms of hopping through a band of localized states, while higher temperature conduction is explained in terms of the thermal excitation of carriers to the band boundaries^2^. Variable Range Hopping (**VRH**) conduction mechanism at low temperatures (300-330 K) is based on localized states near to the Fermi level. According to Mott’s **VRH** approach, at low temperatures, lengthy hops between sites are more frequent than the total of all other ancillary hops. VRH mechanism is described by Mott's phrase as following^2,56–59^:

14 $$\sigma \left(T\right)={\sigma }_{o}^{*}{T}^{-\frac{1}{2}}\mathrm{exp}\left(-\mathrm{A}{T}^{-\frac{1}{4}}\right),$$

15 $$A={T}_{0}^{1/4}=\frac{18{\alpha }^{3}}{{k}_{B}N\left({E}_{f}\right)},$$

16 $${\sigma }_{o}^{*}=3{e}^{2}\gamma {\left(\frac{N\left({E}_{f}\right)}{8\pi \alpha {k}_{B}T}\right)}^{0.5},$$

17 $$\alpha =22.52{\sigma }_{o}^{*}{A}^{2},$$

18 $$N\left({E}_{f}\right)=2.12x {10}^{9}\left({\left({\sigma }_{o}^{*}\right)}^{3}{A}^{2}\right),$$

where $\mathbf{N}\left({\mathbf{E}}_{\mathbf{f}}\right)$ is represents the density of localized states, **T**_**o**_ is degree of disorder and ***γ*** is the Debye frequency (10^13^ Hz)^2^. The hopping distance and hopping energy, which are provided by the following equations, may be derived by concurrently solving Mott’s Eqs. (14), (15), (16), (17), (18)^2^:

**Table 2:** Values of activation energies and Mott parameters for as-deposited PTPA thin films of different thicknesses.

d (nm)	ΔE_σI_	ΔE_σII_	σ_oI_	σ_oII_	T_o_×10^8^ (K)	N(E_f_)×10^18^ (eV^–1^.cm^–3^)	α×10^6^ (cm^–1^)	${R}_{d}$×10^-7^ (cm)	${W}_{E}$(eV)	Rα
100	1.72	1.59	0.471	0.181	11.61	3.48	27.92	6.14	0.295	17.15
190	1.79	1.54	0.342	0.715	10.25	2.85	25.06	6.63	0.286	16.62

19 $${W}_{E}=\left(\frac{3}{4\pi {R}^{3}N\left({E}_{f}\right)}\right),$$

20 $${R}_{d}={\left(\frac{9}{{8\pi \alpha k}_{B}TN\left({E}_{f}\right)}\right)}^{1/4}.$$

**Figure 19:**
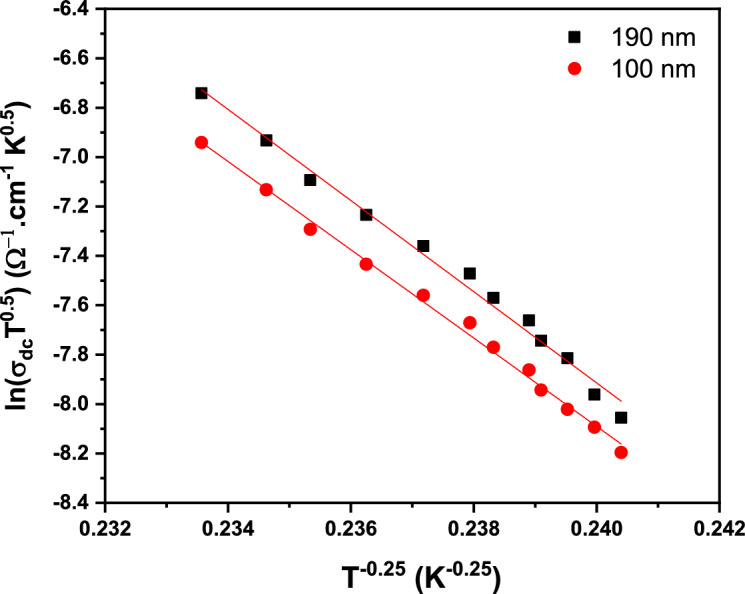
Plot of lnσdc T^0.5^ vs. T^–0.25^ for PTPA thin films.

Figure 19 depicts a plot of ln(σT^1/2^) versus **T**^**1/4**^ for two various thicknesses (100 and 190 nm) from PTPA films. Various parameters, such as ${\varvec{N}}\left({{\varvec{E}}}_{{\varvec{f}}}\right),$ **T**_**0**_, ${{\varvec{R}}}_{{\varvec{d}}}$ and ${{\varvec{W}}}_{{\varvec{E}}}$ are determined using Eqs. (14), (15), (16), (17), (18), (19) and (20) which are presented in Table 2. The hopping distance raises as the film thickness increases, but the hopping energy decreases. Additionally, ${{\varvec{R}}}_{{\varvec{d}}}$ ***> 1*** and ${{\varvec{W}}}_{{\varvec{E}}}$ ***> kT*** are necessary for the **VRH** conduction process^2^ (see Table 2). Thus, in the low-temperature region, the **VRH** model is the domain conduction model.

## Conclusions

In conclusion, PTPA was synthesized and characterized using IR, UV-visible, and NMR methods. Using 1D and 2D NMRs, it was possible to demonstrate the presence of PTPA dye in *Z*-hydrazone form, which was stable due to intramolecular hydrogen bonding in chloroform. Optimized structure and electronic properties (E_HOMO_), (E_LUMO_), and (E_g_) were calculated using DFT and PM6 semiempirical calculations. The spin coating method was utilised to prepare PTPA thin films effectively. XRD patterns for all thicknesses revealed that the PTPA films had a monoclinic phase structure. The crystallite size was estimated inside the nanoscale, demonstrating the nanostructure feature of PTPA thin films. According to Tauc's relationship, indirect ${\mathrm{E}}_{\mathrm{g}1}^{\mathrm{Opt}}$ PTPA films decreased from 2.13 to 1.99 eV, also ${\mathrm{E}}_{\mathrm{g}2}^{\mathrm{Opt}}$ decreased from 3.24 to 2.92 eV as the film thickness increased. The energy band gap measured from CV (2.16 eV) was highly comparable to that predicted from absorption onset ${\mathrm{E}}_{\mathrm{g}1}^{\mathrm{Opt}}$. The dispersion parameters of the reflective index in a normal dispersion zone were investigated using a single oscillator model. Based on a semi-empirical relationship, χ^(3)^ and ${n }^{(2)}$ of PTPA thin films were calculated at photon energies ranging from 0.5 to 4 eV. Computed χ^(3)^ and ${n}^{ (2)}$ values were on the order of ~10^−10^ and10^−9^ esu, respectively at $h\nu \to 0$. The DC electrical conductivity of PTPA thin films followed a pattern comparable to that of organic semiconductors. VRH conduction was dominant at low temperatures. Based on these findings, the PTPA seems to be a particularly efficient OSC, which is useful in advanced photonic applications.

### Supplementary Information

Supplementary Information.

## Data Availability

This manuscript has associated data in a data repository. [Authors’ comment: All data included in this manuscript are available upon request by contacting the corresponding author].

## References

[1] Yang C-M (2023). An organic semiconductor obtained with a low-temperature process for light-addressable potentiometric sensors. Sens. Actuators B Chem..

[2] Dongol M, El-Denglawey A, Elhady AF, Abuelwafa AA (2015). Polaron activation energy of nano porphyrin nickel(II) thin films. Appl. Phys. A.

[3] Li B, Huang H (2023). Furan semiconductors and their application in organic field-effect transistors. Mater. Today Nano.

[4] Tao J, Sun W, Lu L (2022). Organic small molecule semiconductor materials for OFET-based biosensors. Biosens. Bioelectron..

[5] Pei K (2022). Recent advances in molecular doping of organic semiconductors. Surf. Interfaces.

[6] Abuelwafa AA (2023). Impact of phosphorescent dye on the performance and impedance spectroscopy for P3HT: PCBM solar cells. Appl. Phys. A.

[7] Abuelwafa AA, Dongol M, El-Nahass MM, Soga T (2020). Role of platinum octaethylporphyrin (PtOEP) in PCPDTBT: PCBM solar cell performance. J. Mol. Struct..

[8] Soliman HN, Yahia IS (2020). Synthesis and technical analysis of-butyl-3-[(4-chlorophenyl) diazenyl]-4-hydroxy-2H-pyrano [3, 2-c] quinoline-2,5(6H)-dione as a new organic semiconductor: Structural, optical and electronic properties. Dye. Pigm..

[9] Ebead YH, Selim MA, Ibrahim SA (2010). Solvatochromic, acid-base features and time effect of some azo dyes derived from 1, 3-benzothiazol-2-ylacetonitrile: Experimental and semiempirical investigations. Spectrochim. Acta A Mol. Biomol..

[10] Porobić SJ (2020). Absorption and fluorescence spectral properties of azo dyes based on 3-amido-6-hydroxy-4-methyl-2-pyridone: Solvent and substituent effects. Dye. Pigm..

[11] Maliyappa MR, Keshavayya J, Mahanthappa M, Shivaraj Y, Basavarajappa KV (2020). 6-Substituted benzothiazole based dispersed azo dyes having pyrazole moiety: Synthesis, characterization, electrochemical and DFT studies. J. Mol. Struct..

[12] Fradj AB, Boubakri A, Hafiane A, Hamouda SB (2020). Removal of azoic dyes from aqueous solutions by chitosan enhanced ultrafiltration. Result Chem..

[13] Tong X, Zhao Y (2009). Multiple photochemical processes in liquid crystalline azo dye-doped liquid crystals. Chem. Mater..

[14] Raposo MMM, Castro MCR, Fonseca AMC, Schellenberg P, Belsley M (2011). Design, synthesis, and characterization of the electrochemical, nonlinear optical properties, and theoretical studies of novel thienylpyrrole azo dyes bearing benzothiazole acceptor groups. Tetrahedron.

[15] Coelho PJ, Castro MCR, Fonseca AMC, Raposo MMM (2012). Photoswitching in azo dyes bearing thienylpyrrole and benzothiazole heterocyclic systems. Dye. Pigm..

[16] Alsoghier HM (2019). NMR spectroscopic, linear and non-linear optical properties of 1,3-benzothiazol-2-yl-(phenylhydrazono)acetonitrile (PTPA) azo dye. J. Mol. Struct..

[17] Bagheri Novir S, Hashemianzadeh SM (2015). Density functional theory study of new azo dyes with different π-spacers for dye-sensitized solar cells. Spectrochim. Acta A Mol. Biomol..

[18] Solomon RV, Jagadeesan R, Vedha SA, Venuvanalingam P (2014). A DFT/TDDFT modelling of bithiophene azo chromophores for optoelectronic applications. Dye. Pigm..

[19] Gouda MA, Eldien HF, Girges MM, Berghot MA (2016). Synthesis and antitumor evaluation of thiophene based azo dyes incorporating pyrazolone moiety. J. Saudi Chem. Soc..

[20] Badahdah KO (2008). Arylhydrazononitriles as building blocks in heterocyclic synthesis: Synthesis of new benzothiazolyl-1,2,3-triazole amines and-1,2,3-triazol-4-yl-1,3,4-thiadiazole-5-ylamines. Heterocycles.

[21] Rida SM (2005). Synthesis of some novel benzoxazole derivatives as anticancer, anti-HIV-1 and antimicrobial agents. Eur. J. Med. Chem..

[22] Shawali AS (2010). Synthesis and tautomerism of aryl- and hetaryl-azo derivatives of bi- and tri-heterocycles. J. Adv. Res..

[23] Towns AD (1999). Developments in azo disperse dyes derived from heterocyclic diazo components. Dye. Pigm..

[24] Lyčka A, Webb GA (1993). Multinuclear NMR of azo dyestuffs. Annu. Rep. NMR Spectrosc..

[25] Lyčka A (2017). 4-Carboxyl-2,6-dinitrophenylazohydroxynaphthalenes tautomerism NMR re-explained. Dye. Pigm..

[26] Babür B (2015). Phenylazoindole dyes 3: Determination of azo-hydrazone tautomers of new phenylazoindole dyes in solution and solid state. J. Mol. Struct..

[27] Mohammadi A, Safarnejad M (2014). Synthesis, structural characterization and tautomeric properties of some novel bis-azo dyes derived from 5-arylidene-2, 4-thiazolidinone. Spectrochim. Acta A Mol. Biomol..

[28] Odabaşoğlu M, Albayrak Ç, Özkanca R, Aykan FZ, Lonecke P (2007). Some polyhydroxy azo–azomethine derivatives of salicylaldehyde: Synthesis, characterization, spectroscopic, molecular structure and antimicrobial activity studies. J. Mol. Struct..

[29] Zhang L, Cole JM, Liu X (2013). Tuning. Solvatochromism of azo dyes with intramolecular hydrogen bonding in solution and on titanium dioxide nanoparticles. J. Phys. Chem. C.

[30] Antonín L, Josef J, Aleš C (1990). 15N, 13C and 1H NMR spectra of the 2:1 cobalt(III) complexes of some azo dyes. Magn. Reson. Chem..

[31] Kurasawa Y (1994). Substituent effects on the tautomer ratios between the hydrazone imine and diazenyl enamine forms in side-chained quinoxalines. J. Heterocycl. Chem..

[32] Gutierrez JA, Falcone RD, Silber JJ, Correa NM (2010). Role of the medium on the C343 inter/intramolecular hydrogen vond interactions. An absorption, emission, and 1HNMR investigation of C343 in benzene/n-heptane mixtures. J. Phys. Chem. A.

[33] Goutev N, Matsuura H (2001). Hydrogen bonding in chloroform solutions of ethylenedioxy ethers. Spectroscopic evidence of bifurcated hydrogen bonds. J. Phys. Chem. A.

[34] Raposo MMM, Castro MCLR, Belsley M, Fonseca AMC (2011). Push-pull bithiophene azo-chromophores bearing thiazole and benzothiazole acceptor moieties: Synthesis and evaluation of their redox and nonlinear optical properties. Dye. Pigm..

[35] Laugier, J. & Bochu, B. LMGP-suite of programs for the interpretation of X-ray experiments, ENSP/laboratories des materiaux et du genie physique BP46.38042, Saint Martin d’Heres, France (2000).

[36] Shirley R (1999). The CRYSFIRE System for Automatic Powder Indexing: User’s Manual.

[37] Abuelwafa AA, El-Denglawey A, Dongol M, El-Nahass MM, Soga T (2015). Influence of annealing temperature on structural and optical properties of nanocrystalline platinum octaethylporphyrin (PtOEP) thin films. Opt. Mater..

[38] Abuelwafa AA, Alsoghier HM, Elnobi S, Dongol M, Soga T (2021). Quantum computational, linear and non-linear optical properties of spin-coated nickel (II)-tetraphenylporphyrin/FTO thin films. Optik.

[39] Gami F, Guizani I, Sebak MA, Abuelwafa AA, Mostafa MM (2022). Investigation of structural, optical and electrical properties of PCBM/ZnOEP thin films. Opt. Mater..

[40] Abuelwafa AA, Elnobi S, Yamada I, Shibata N, Soga T (2022). Studying linear and nonlinear optical properties of trifluoroethoxy-coated zinc phthalocyanine (4TFEO) 4-ZnPc) thin films. Opt. Mater..

[41] Elnobi S, Abd El-sadek MS, Yahia IS, Zahran HY, Abuelwafa AA (2022). Correlation between structural, morphological, and optical properties of spin-coated poly (2,5-di(hexyloxy) cyanoterephthalylidene) (CN-PPV) thin films. J. Mater. Sci. Mater. Electron..

[42] El-Zaidia EFM (2023). Effect of film thickness on structural, electrical and optical properties of amorphous boron subphthalocyanine chloride thin film. Opt. Mater..

[43] Denga X (2022). Terpolymerization strategy to achieve high-efficient organic solar cells via constructing D1-A-D1-D2-type polymer donors. Chem. Commun..

[44] Wasly HS, Abd El-sadek MS, Elnobi S, Abuelwafa AA (2022). Morphological, structural, and optical properties of flexible Tin Oxide (II) thin film via thermal evaporation technique. Eur. Phys. J. Plus.

[45] Abuelwafa AA, Abd El-sadek MS, Elnobi S, Soga T (2021). Effect of transparent conducting substrates on the structure and optical properties of Tin (II) oxide (SnO) thin films: Comparative study. Ceram. Int..

[46] Wemple H, DiDomenico M (1971). Behavior of the electronic dielectric constant in covalent and ionic materials. Phys. Rev. B.

[47] Matiur MD, Abuelwafa AA, Kato S, Kishi N, Soga T (2021). A comparative study on optical properties of BiOI, Bi_7_O_9_I_3_ and Bi_5_O_7_I materials. Opt. Mater..

[48] Ticha H, Tichy L (2023). Remark on the correlation between the refractive index and the optical band gap in some crystalline solids. Mater. Chem. Phys..

[49] Ebied MS (2022). Structural and optical properties of nanocrystalline 3-(2-benzothiazolyl)-7-(diethylamino) coumarin (C6) thin films for optoelectronic application. J. Electron. Mater..

[50] Sheik-Bahae M, Hutchings DC, Hagan DJ, Stryland EWV (1991). Dispersion of bound electron nonlinear refraction in solids. IEEE J. Quantum Electron..

[51] Sheik-Bahae M, Hagan DJ, Stryland EWV (1990). Dispersion and band-gap scaling of the electronic Kerr effect in solids associated with two-photon absorption. Phys. Rev. Lett..

[52] Attia AA, El-Barry AMA, El-Shazly EAA, El-Deen LMD (2018). Studies on structural and optical properties of thermally evaporated nanocrystalline thin films of meso-Tetraphenylporphyrin manganese (III) chloride. J. Lumin..

[53] Nasher MA, Youssif MI, El-Ghamaz NA, Zeyada HM (2019). Linear and nonlinear optical properties of irradiated toluidine blue thin films. Optik.

[54] Pandey M, Joshi GM, Deshmukh K, Ghosh NN, Raj NAN (2015). Electrical conductivity, optical properties and mechanical stability of 3, 4, 9, 10-perylenetetracarboxylic dianhidride based organic semiconductor. J. Phys. Chem. Solids.

[55] Kobayashi Y, Suzuki A, Yamada Y, Saigo K, Shibue T (2010). Synthesis, characterization, and dc conductivity of hydrogen-bonding dibenzotetrathiafulvalene (DBTTF) based salts. Synth. Met..

[56] El-Ghamaz NA, El-Bindary AA, El-Boz RA (2017). Electrical and optical properties of new azo dyes derived from m-aminophenol. Synth. Met..

[57] Leontie L (2018). Electric and optical properties of some new functional lower-rimsubstituted calixarene derivatives in thin films. Appl. Phys. A.

[58] Maddison DS, Tansley TL (1992). Variable range hopping in polypyrrole films of a range of conductivities and preparation methods. J. Appl. Phys..

[59] El-Ghamaz NA, Diab MA, El-Sonbati AZ, Salem OLDC (2011). Electrical conductivity and conduction mechanism of some azo sulfonyl quinoline ligands and uranyl complexes. Spectrochim. Acta A Mol. Biomol..

