# Supplementary material for: A novel organic semiconductor 4-phenylthiazol-2-yl-(phenylhydrazono) acetonitrile (PTPA) thin films: synthesis, optical and electrical properties
Source: Sci Rep. 2023 Aug 10;13:12973. doi: 10.1038/s41598-023-39027-3 (PMC10415267; doi:10.1038/s41598-023-39027-3)
Supplement: Supplementary file 1 — Supplementary Information. [file 41598_2023_39027_MOESM1_ESM.docx]

**Supplementary Material**

**A Novel Organic Semiconductor 4-phenylthiazol-2-yl-(phenylhydrazono) acetonitrile (PTPA) Thin Films: Synthesis, Optical and Electrical properties**

Amr Attia Abuelwafa^a*^, Sahar Elnobi^b^, M. A. Santos^c^, H. M. Alsoghier^d^

^a^ Nano & Thin Film Lab, Physics Department, Faculty of Science, South Valley University, Qena 83523, Egypt.

^b^ Chemistry Department, Faculty of Science, South Valley University, 83523 Qena, Egypt.

^c^ Centro de Química Estrutural, Instituto Superior Técnico, Universidade de Lisboa, Av. Rovisco Pais 1, 1049-001 Lisboa, Portugal

^d^ Physics Department, Faculty of Science, South Valley University, Qena 83523, Egypt.

***^*^Corresponding author***

****e-mail:*** [***Amr.abuelwafa@sci.svu.edu.eg***](mailto:*Amr.abuelwafa@sci.svu.edu.eg) ***(*Amr Attia Abuelwafa*)***


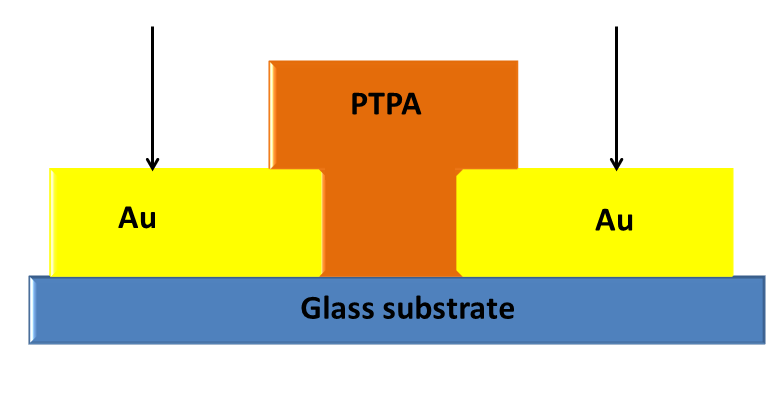


**Scheme S1.** Schematic illustration of the cross-section specimens' shape for electrical conductivity testing.

**Figure S1:** ^13^C NMR spectrum of the 4-phenylthiazol-2-yl-(phenylhydrazono) acetonitrile (**PTPA**) azo dye in CDCl_3_.

**Figure S2:** ^13^C DEPT-135 NMR spectrum of the dye (**PTPA**) in CDCl_3_.

.


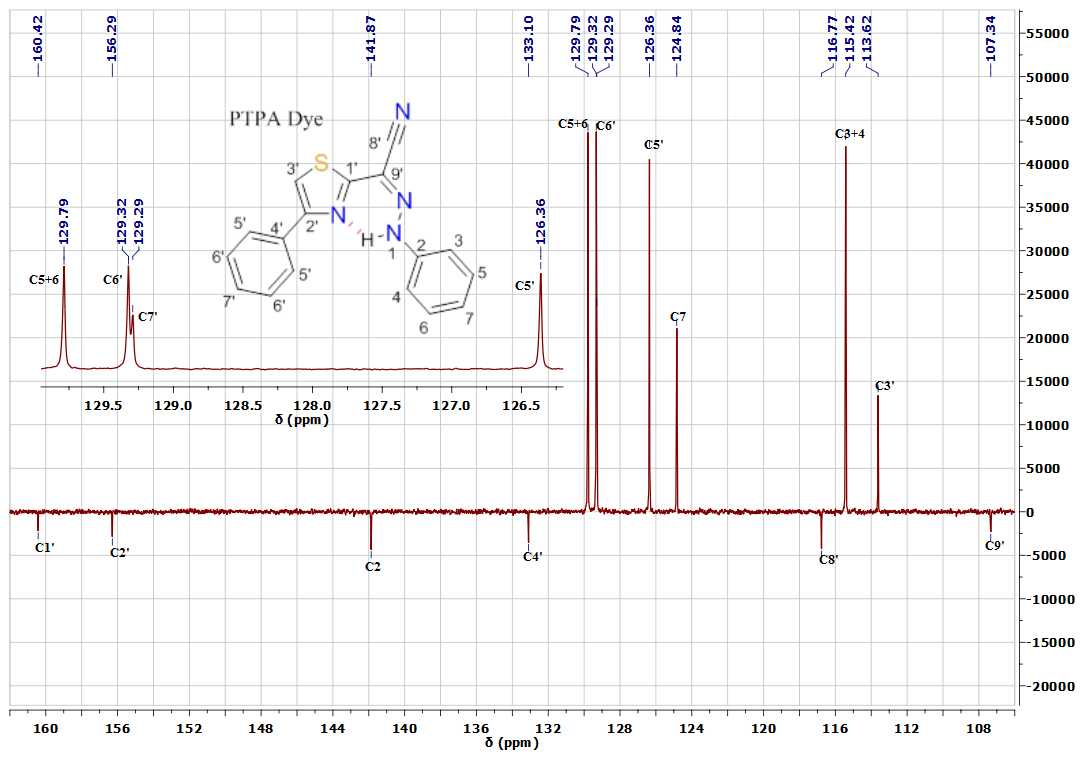


**Figure S3:** ^13^C APT NMR spectrum of the dye (PTPA) in CDCl_3_.


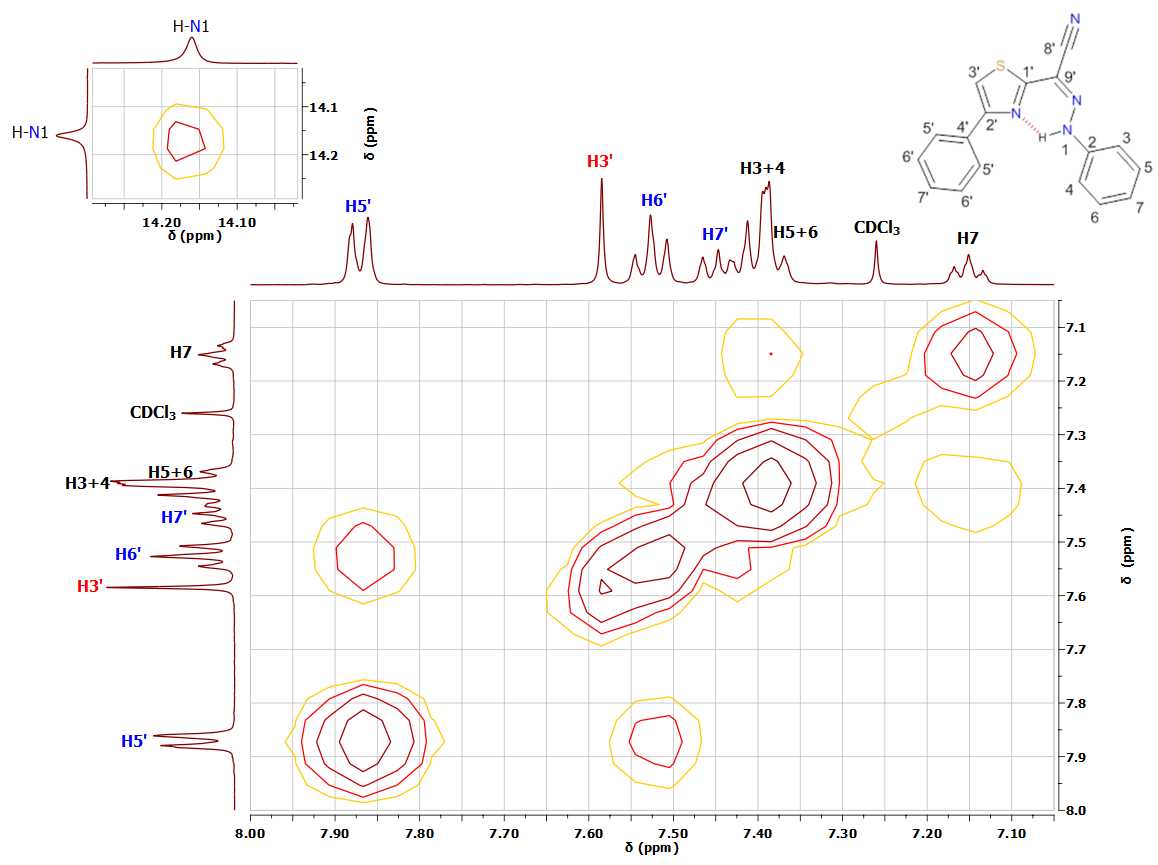


**Figure S4:** ^1^H-^1^H COSY NMR spectrum of the dye (**PTPA**) in CDCl_3_.

**
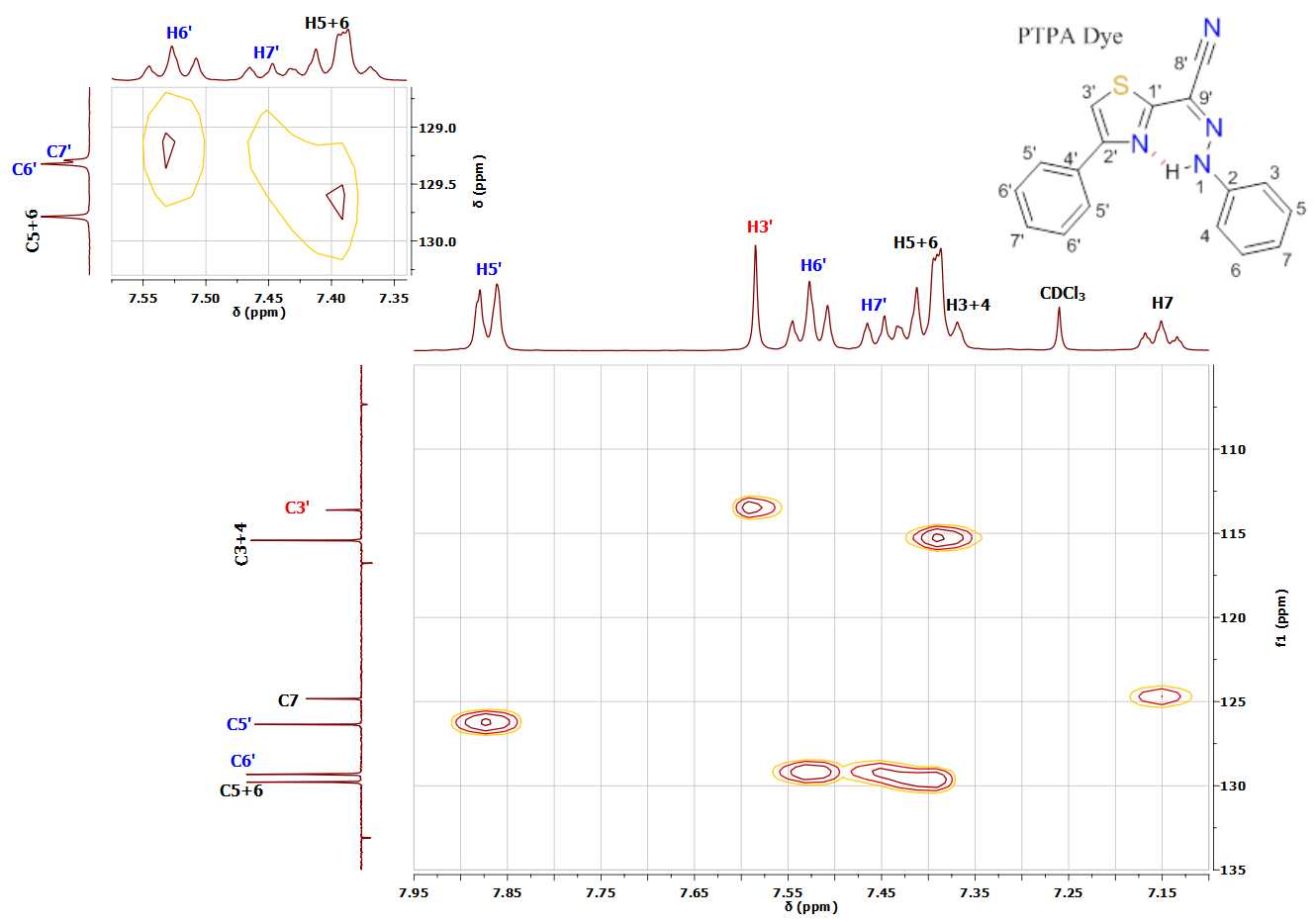
**

**Figure S5:** ^1^H-^13^C HSQC NMR spectrum of the dye (**PTPA**) in CDCl_3_.

**Table** S**1:** ^1^H and ^13^C chemical shifts (ppm) and protons coupling constants (J) of 4-phenylthiazol-2-yl-(phenylhydrazono) acetonitrile (PTPA) azo dye in CDCl_3_.

| H/C No. | **PTPA** | |
| --- | --- | --- |
|  | δ(^1^H) | δ(^13^C) |
| 1' | - | 160.43 |
| 2' | - | 156.30 |
| 3' | 7.58 (s, 1H) | 113.62 |
| 4' | - | 133.10 |
| 5' | 7.87 (d, J = 7.3 Hz, 2H) | 126.36 |
| 6' | 7.53 (t, J = 7.5 Hz, 2H) | 129.32 |
| 7' | 7.45 (t, J = 7.3 Hz, 1H), | 129.29 |
| 8' | - | 116.77 |
| 9' | - | 107.34 |
| 2 | - | 141.87 |
| 3,4 | 7.38 (d, J = 7.1 Hz, 2H) | 115.42 |
| 5,6 | 7.42 (d, J = 7.2 Hz, 2H) | 129.79 |
| 7 | 7.15 (t, J = 6.8 Hz, 1H) | 124.84 |
| 1N-H hydrazone | 14.16 (s, 1H) | - |


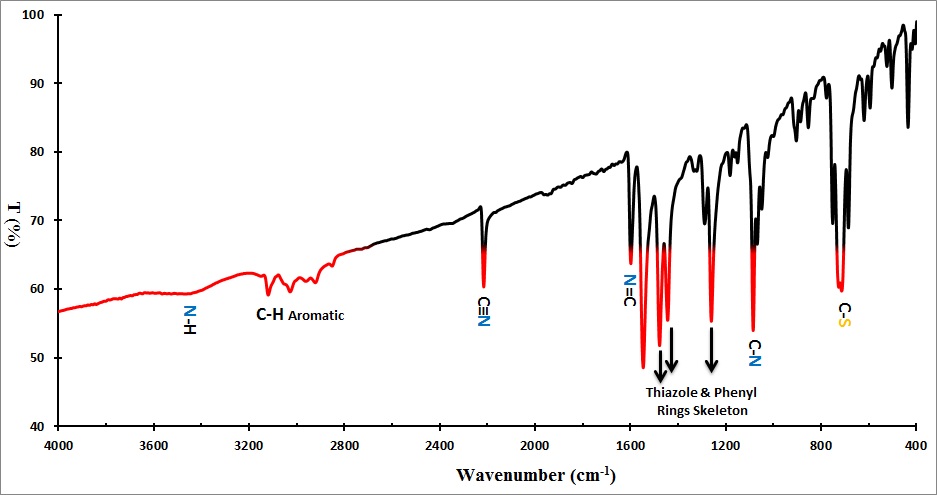


**Figure S6:** FT-IR of the dye (**PTPA**) powder.
